# Obstetrics and Gynecology Modified Delphi Survey for Entrustable Professional Activities: Quantification of Importance, Benchmark Levels, and Roles in Simulation-based Training and Assessment

**DOI:** 10.7759/cureus.3051

**Published:** 2018-07-25

**Authors:** Milena Garofalo, Rajesh Aggarwal

**Affiliations:** 1 Obstetrics and Gynaecology, Medicine/McGill University Health Centre, Montreal, CAN; 2 Surgery, Strategic Business Development/Thomas Jefferson University and Jefferson Health, Philadelphia, USA

**Keywords:** simulation, competency-based education, obstetrics, gynecology, epa

## Abstract

Objective

Competency-based medical education (CBME) is playing a central role in physicians’ training. It focuses on competencies, measured by entrustable professional activities (EPAs). The aim of this survey is threefold for each EPA to (1) quantify the importance for Obstetrics and Gynecology (OBGYN) residency training; (2) set benchmarks; (3) identify the importance of simulation-based training (SBT).

Methods

The EPAs were defined based on a review of five OBGYN curricula. Two rounds of a modified Delphi via online questionnaire were performed from January to March, 2017. Experts were North American OBGYN program directors. Using a Likert scale, they rated the importance of each EPA for residency training, identified benchmark levels of competence, and roles of simulation. Consensus was defined as ≥80% agreement.

Results

Item analysis yielded 15 EPAs. Survey response rate was 17.47% (40 out of 229) for part 1 and 6.55% for part 2 (15 out of 229). All experts rated the importance of each EPA for residency training as “moderately important” or “absolutely essential”. For benchmarking, experts agreed with a stepwise increase in the level of competence, dependent on residency stage. Two EPAs, “Gynecological Technical Skills & Procedures” and “High-Risk Childbirth”, reached consensus (rating 4 or 5) for simulation.

Conclusion

CBME requires EPAs and benchmarks for each residency stage. Simulation will become a valuable tool in this model. However, experts remain neutral about its role, except for technical skills. An OBGYN curriculum based on predefined EPAs, benchmarks, and adequate assessment tools, including simulation, needs to be further explored for CBME to be successful.

## Introduction

Competency-based medical education (CBME) plays a central role in today’s training of physicians [1-4]. The concept of CBME is to ensure stages of training, with a focus on specific acquisition of competencies, is measured by entrustable professional activities (EPAs) and milestones [5-6]. Competencies are defined as “the synthesis of knowledge, skills, and attitudes that are reflected in professional activities” [7]. The concept of EPA was introduced by Ten Cate & Scheele in 2007 in order to “bridge the gap between competency-driven education and clinical practice” because “competencies are intertwined in complex ways that make them less explicit and measurable” [3,5]. An EPA is a specific task or activity that can be ‘entrusted’ to a person once sufficient competence has been achieved. EPAs represent day to day work; they are executable, observable, and measurable entities and can be the focus of assessment [8]. A milestone is defined as “an observable marker of an individual’s ability” [6,9]. Typically each EPA integrates multiple competencies and milestones [5-6,8-9].

Based on a review of national Obstetrics and Gynecology (OBGYN) residency curricula from five countries (Australia, Canada, Netherlands, UK, and the USA), it was concluded that all curricula have or will be integrating CBME into their training programs [10]. Nonetheless, there is a need to develop adequate training and assessment tools, including simulation, to ultimately deliver competent physicians, capable of unsupervised practice. Although some programs within Canada have already started piloting CBME and EPAs, [11] the national Canadian “Competence By Design” (CBD) program is expected to roll-out officially for OBGYN in 2019 [9].

Assessment of clinical competence can be done in a variety of ways based on four levels, as shown in Miller’s pyramid [12]. The lower cognitive levels “knows” and “knows how”, typically assessed by written and oral exams, do not ensure that knowledge is transferred into competence. This contrasts with the higher behavioural levels of Miller’s pyramid, “shows how” and “does”, where competence can be demonstrated. Examples include simulation, Objective Structured Clinical Examination and Assessment of Technical Skills (OSCE/OSATS) and workplace based assessments (WBAs). Simulation may serve as an additional or alternative outlet to WBAs since these remain a challenge in everyday clinical practice. Simulation will thus be a valuable tool in the training and assessment of residents in the CBD program, allowing direct observation of many technical and non-technical skills. It allows the opportunity to improve performance by mimicking reality in a safe learning environment [13-15].

The aims of this Delphi survey are threefold: first, quantify the importance of each pre-defined EPA with respect to OBGYN residency training; second, set benchmark levels for each EPA; and third, identify the importance of simulation-based training and assessment for each EPA. The ultimate goal is to provide a framework for building a national curriculum based on predefined EPAs and benchmarks levels at each stage of residency training, and develop training and assessment tools using simulation. This was based on expert consensus using a modified Delphi consensus method.

## Materials and methods

**Figure 1:**
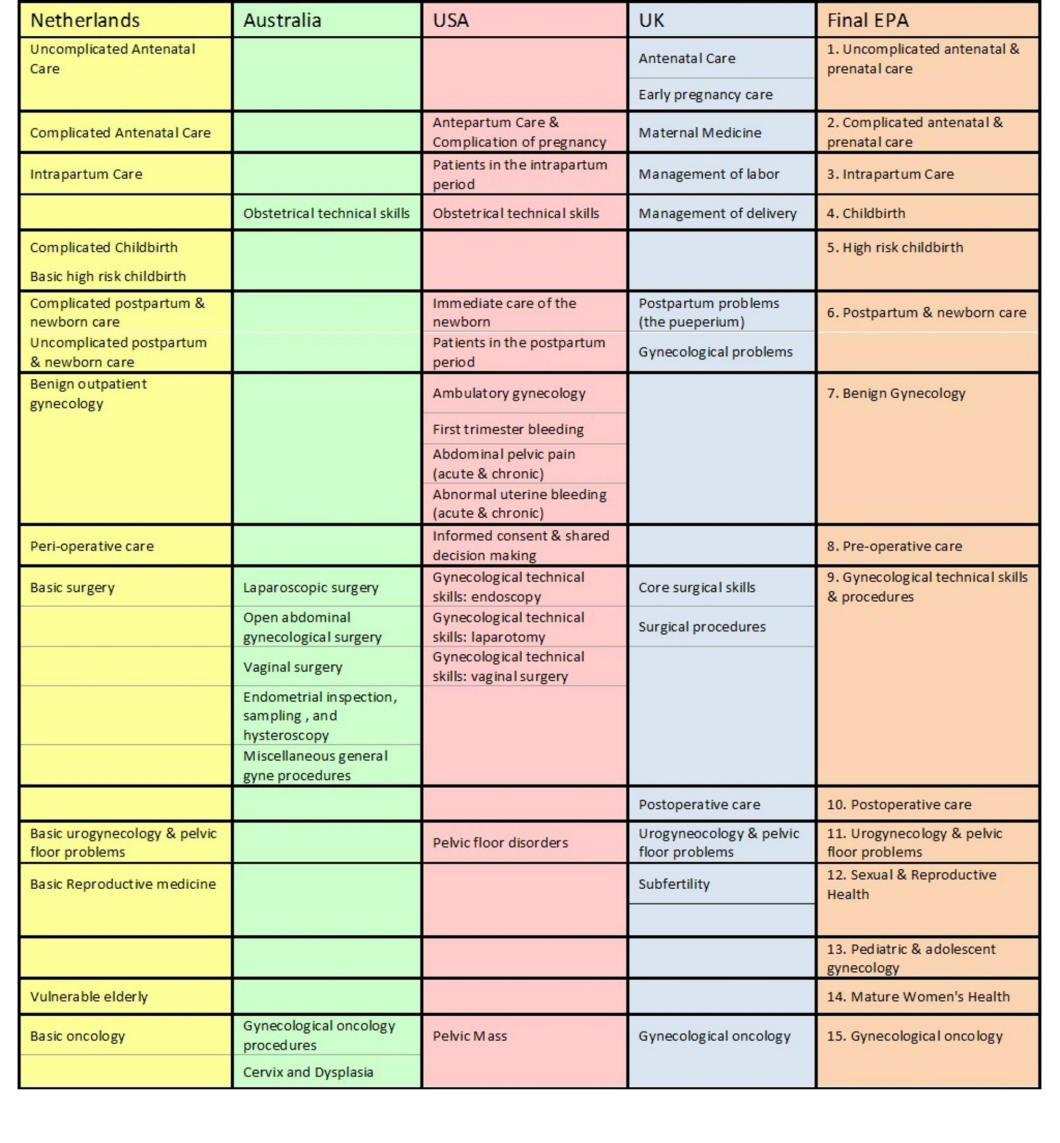
Composite List of Final Entrustable Professional Activities (EPA) Based on Review of Literature

**Figure 2:**
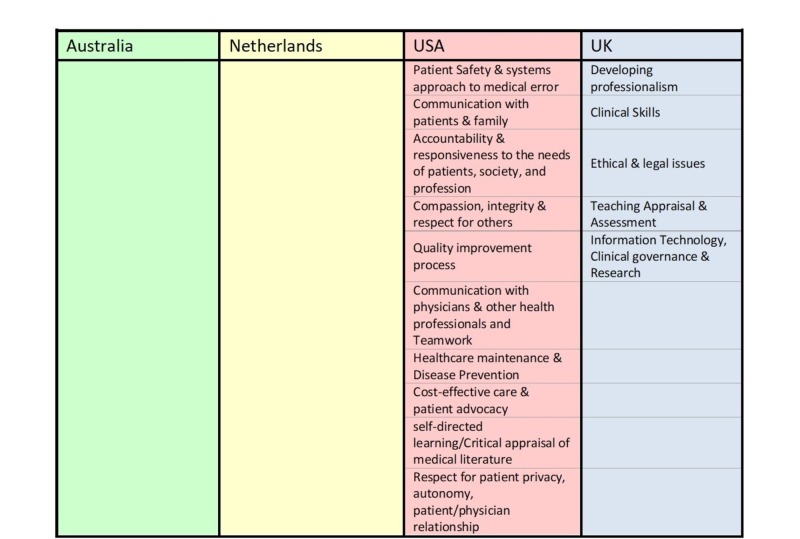
Discarded List of Entrustable Professional Activities (EPA) Representing General Characteristics

We defined the individual items (EPAs) used in the survey based on a review of five international OBGYN curricula [10]. This process was undertaken with the engagement of a medical educator and an OBGYN clinician. We established that the EPAs defined in the Dutch curriculum would be the framework, since it most closely resembles the Canadian model [7] (Figure 1). We excluded potential EPAs that constituted general characteristics that could be part of the description and milestones for other more specific OBGYN EPAs (e.g., ethical and legal issues, patient safety, communication) (Figure 2). We performed two rounds of a modified Delphi survey via online questionnaire through “Survey Monkey” to (1) rate the importance of each EPA for residency training, (2) rate the importance of simulation-based training and assessment for each EPA and (3) identify benchmark levels of competence, for each specified stage of residency training. Experts in the study were OBGYN residency program directors across Canada and the United States (US), who received an email request to participate in an online questionnaire. Experts were asked to rate the importance of each EPA for residency training based on a Likert scale from 1 to 5:

1.     Not at all important

2.     Minimal importance

3.     Neutral

4.     Moderately essential

5.     Absolutely essential

They were then asked to rate the importance of simulation-based training and assessment for each EPA, using the same Likert scale. Finally, the last part of the survey set out to identify benchmark levels of competence, for each specified stage of residency training. We defined the stages of residency training using Eraut’s summary of Dreyfus and Dreyfus’ model of skill acquisition [16-17] and the US “Milestone Project” levels of observed behavior [18]: 

Stage 1: Novice

Stage 2: Advanced Beginner

Stage 3: Competent

Stage 4: Proficient

We can parallel these to the 4 stages of residency in the Canadian CBD continuum: transition to discipline, foundations of discipline, core of discipline, and transition to practice [19]. We did not include the fifth stage of expert/master as it is expected to reach this stage later in one’s career, during continuing professional development. We defined the levels of competence using the competency levels in the Dutch OBGYN curriculum [7], mapped to the Canadian Model and originally based on the instructional scaffolding theory of the zone of proximal development (ZPD) in educational psychology [3,6,20-22]. This theory describes three zones: what a learner can do; what a learner can do with guidance (ZPD); and what a learner can do unaided.

Level 1: Modeling - has knowledge of

Level 2: Scaffolding - performs with full supervision

Level 3: Fading - performs with limited supervision

Level 4: Entrustment - performs without supervision

Level 5: Able to supervise/teach others

We collected and analyzed responses from this panel of experts. We defined consensus as ≥80% agreement for a rating of 4 (moderately essential) and 5 (absolutely essential). Two rounds of the Delphi survey took place. In the second round, experts were told the mean responses from the first round. Ethics approval was granted by the Institutional Review Board (IRB) of the McGill University Faculty of Medicine. This is by itself acceptable for approaching US-based participants as the McGill IRB acts in accordance with the US Code of Federal Regulations and hold an Inter-institutional Agreement.

## Results

The item analysis based on the review of five international OBGYN curricula yielded a list of 15 EPAs:

1.  Uncomplicated Antenatal & Prenatal Care

2.  Complicated Antenatal & Prenatal Care

3.  Intrapartum Care

4.  Childbirth

5.  High Risk Childbirth

6.  Postpartum & newborn care

7.  Benign Gynecology

8.  Gynecological Technical Skills & Procedures

9.  Pre-operative care

10.  Postoperative Care

11.  Mature Women's Health

12.  Gynecological oncology

13.  Urogynecology & Pelvic Floor Problems

14.  Pediatric & Adolescent Gynecology

15.  Sexual & Reproductive Health

**Figure 3:**
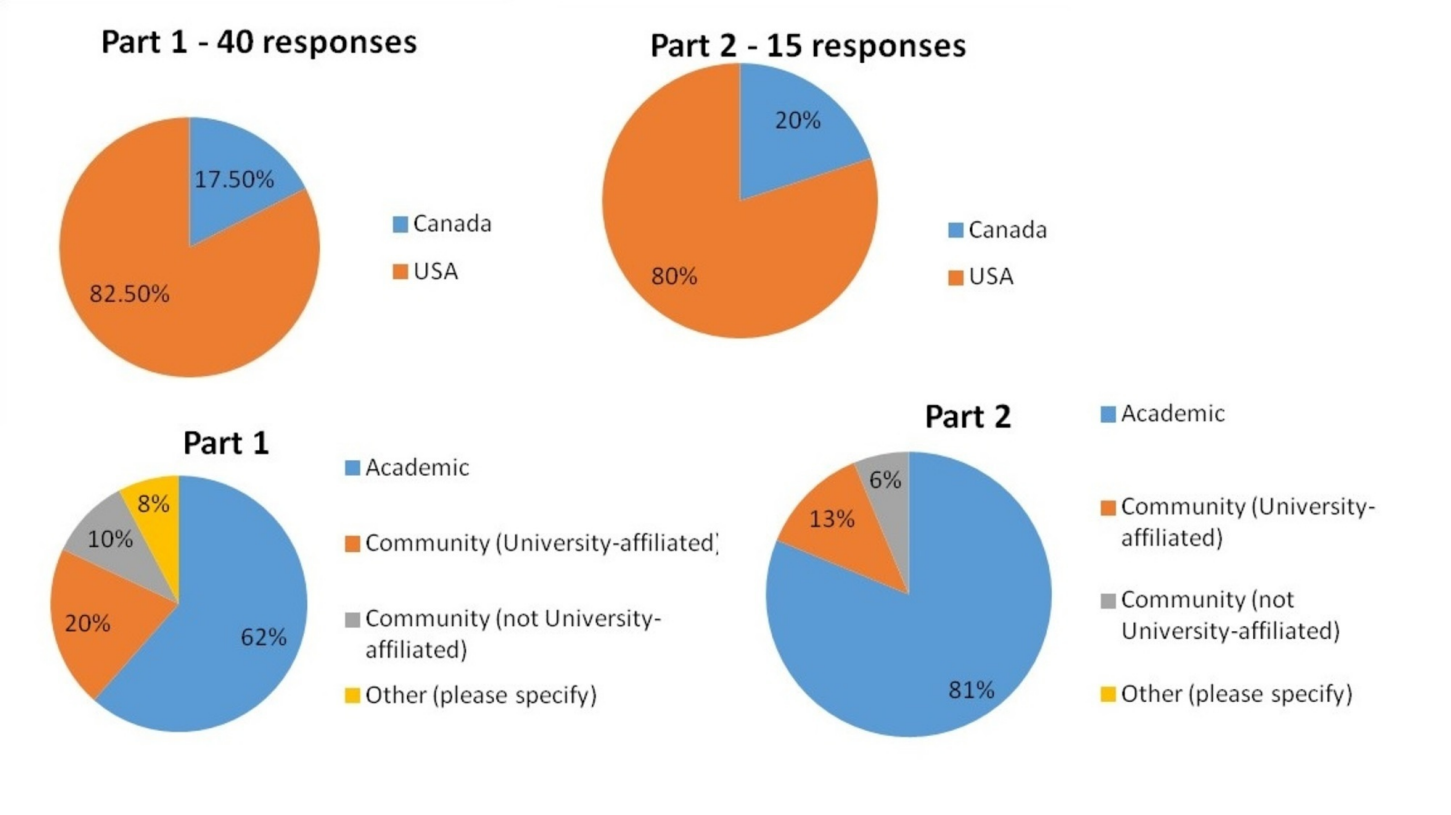
Delphi Survey Demographics

The overall response rates for the survey were 17.47% (40 out of 229) for part 1 and 6.55% for part 2 (15 out of 229). Of those that participated in part 1, 17.5% were Canadian program directors and 82.5% were American, giving a Canadian response rate of 38.89% and an American response rate of 15.64%. In part 2, 20% were Canadian program directors giving response rates of 16.67% and 80.00% were American with response rates of 5.69%. Most experts were from academic or university-affiliated community hospitals (Figure 3).

The majority of experts (84.38% to 100.00%) experts rated the importance of each EPA for residency training as “moderately important” (4) or “absolutely essential” (5) (Table 1). There was much more variability in the responses when it came to rating the importance for each EPA for simulation-based training and assessment. Only two EPAs had a consensus of ≥80% for rating of 4 or 5 for both simulation based training and assessment: “Gynecological Technical Skills & Procedures” and “High Risk Childbirth”. The following highest rated EPA was “Childbirth” at 71.43%-72.41% (Tables 2-3). Experts remained “neutral” (mean of 3, Standard Error of the Mean (SEM) 0.17 and 0.15) for all other EPAs, with regards to their importance in simulation as an educational modality for this EPA. 

**Figure 4:**
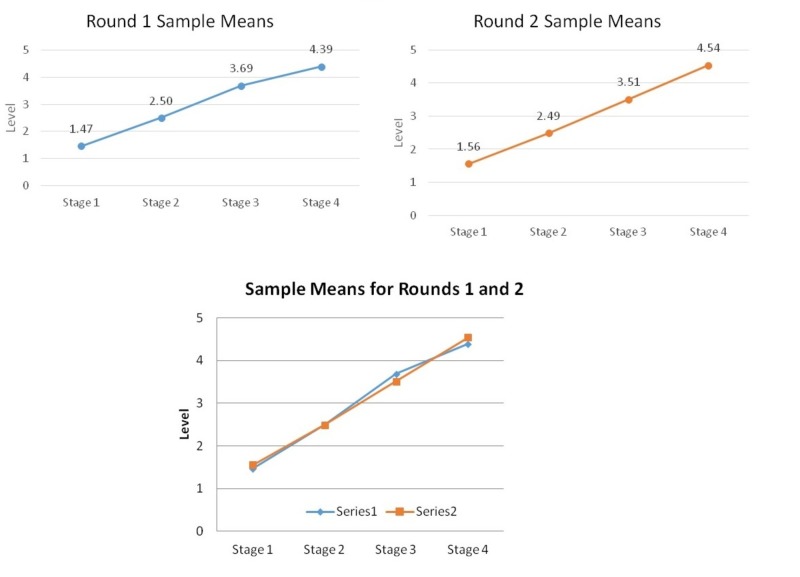
Benchmarks for each Entrustable Professional Activity (EPA) – “For each stage of residency training, what is the expected overall mean level of competence?”

The last part of or survey set out to determine benchmark levels for each EPA at each stage of residency. At Stage 1, the novice learner is expected to be modelling (level 1) or scaffolding (level 2) (mean 1.47-1.56, SEM 0.07-0.08), with 90.48% to 100.00% consensus. At Stage 2, the advanced beginner is expected to be scaffolding (level 2) or fading (level 3) (mean 2.49-2.50, SEM 0.09), with 91.67% to 100.00% consensus. At Stage 3, the competent learner is expected to be fading (level 3) and be entrusted (level 4) for most EPAs (mean 3.51-3.69, SEM 0.10-0.11), with 83.33% to 100.00% consensus. There were two exceptions in Round 1 where consensus was not met: “Postpartum and newborn care” (77.78%) and “Post-operative care” (77.78%), suggesting that for these EPAs, the learner may reach level 5 at an earlier stage in training. At Stage 4, the proficient learner is expected to be entrusted (level 4) and can supervise and teach others (level 5) for most EPAs (mean 4.39-4.54, SEM 0.09-0.11), with 80.00% to 100.00% consensus (Table 2) (Figure 4).

**Table 1:** Importance of each Entrustable Professional Activity (EPA) for Residency Training

EPA	Round 1		Round 2	
	Mean	% 4 or 5	Mean	% 4 or 5
Uncomplicated Antenatal & Prenatal Care	4.94	100.00	5.00	100.00
Complicated Antenatal & Prenatal Care	4.84	100.00	4.93	100.00
Intrapartum Care	4.94	100.00	5.00	100.00
Childbirth	4.91	100.00	5.00	100.00
High Risk Childbirth	4.72	93.75	5.00	100.00
Postpartum & newborn care	4.5	93.75	4.86	100.00
Benign Gynecology	4.91	100.00	5.00	100.00
Gynecological Technical Skills & Procedures	4.97	100.00	4.93	100.00
Pre-operative care	4.78	93.75	5.00	100.00
Postoperative Care	4.88	100.00	5.00	100.00
Mature Women's Health	4.72	96.88	4.86	100.00
Gynecological oncology	4.41	93.75	4.57	100.00
Urogynecology & Pelvic Floor Problems	4.44	93.75	4.57	100.00
Pediatric & Adolescent Gynecology	4.13	84.38	4.29	100.00
Sexual & Reproductive Health	4.55	90.32	4.64	100.00

**Table 2:** Importance of each Entrustable Professional Activity (EPA) for Simulation-based Training

EPA	Round 1		Round 2	
	Mean	% 4 or 5	Mean	% 4 or 5
Uncomplicated Antenatal & Prenatal Care	2.14	13.79	2.43	14.29
Complicated Antenatal & Prenatal Care	2.79	31.03	2.57	14.29
Intrapartum Care	3.55	58.62	3.31	61.54
Childbirth	3.72	72.41	3.57	71.43
High Risk Childbirth	4.00	75.86	4.14	92.86
Postpartum & newborn care	2.59	31.03	3.00	35.71
Benign Gynecology	2.83	34.48	2.92	46.15
Gynecological Technical Skills & Procedures	4.41	89.66	4.57	100.00
Pre-operative care	2.34	10.34	2.36	14.29
Postoperative Care	2.72	34.42	2.50	14.29
Mature Women's Health	2.24	6.90	2.29	14.29
Gynecological oncology	2.83	24.14	2.57	21.43
Urogynecology & Pelvic Floor Problems	3.17	37.93	3.29	42.86
Pediatric & Adolescent Gynecology	2.72	24.14	2.79	28.57
Sexual & Reproductive Health	2.79	31.04	2.79	28.57

**Table 3:** Importance of each Entrustable Professional Activity (EPA) for Simulation-based Assessment

EPA	Round 1		Round 2	
	Mean	% 4 or 5	Mean	% 4 or 5
Uncomplicated Antenatal & Prenatal Care	2.41	17.24	2.50	14.29
Complicated Antenatal & Prenatal Care	2.93	34.48	2.64	14.29
Intrapartum Care	3.48	62.07	3.15	61.54
Childbirth	3.72	72.41	3.64	71.43
High Risk Childbirth	3.90	75.86	4.07	85.71
Postpartum & newborn care	2.66	34.48	3.00	38.46
Benign Gynecology	2.90	37.93	2.79	35.71
Gynecological Technical Skills & Procedures	4.21	89.66	4.07	85.71
Pre-operative care	2.34	10.34	2.50	14.29
Postoperative Care	2.76	34.48	2.50	14.29
Mature Women's Health	2.38	13.79	2.21	7.14
Gynecological oncology	2.83	24.14	2.50	14.29
Urogynecology & Pelvic Floor Problems	3.17	41.38	3.21	35.71
Pediatric & Adolescent Gynecology	2.62	17.24	2.79	28.57
Sexual & Reproductive Health	2.69	27.59	2.79	28.57

## Discussion

This Delphi consensus determined the importance of OBGYN EPAs with respect to residency training, set benchmarks for each stage of residency, and identified the role of simulation in both training and assessment. First, a task analysis yielded a list of 15 EPAs for OBGYN based on a review of international curricula. Second, this list was virtually presented as a questionnaire to a North American panel of OBGYN residency program directors and validated through consensus. For the benchmarks, the majority of experts agreed that for each EPA there is a stepwise increase in the level of competence depending on the stage of residency. Most of the panel in this study were neutral about the role of simulation in OBGYN, except for the learning of surgical and procedural skills. Perhaps this may be explained by their current experience and more extensive use of simulation in these specific areas of practice. However, the value of simulation for residency training extends beyond that of purely technical skills. There is a growing body of literature showing its use in team-based training, communication, and crisis-resource management [23-26]. The EPAs listed in this study may have been too broad for the experts to see the potential or the experts may simply be unfamiliar with the different simulations available for medical education. This might make it difficult to confidently include or exclude simulation as a relevant modality. Another survey, outlining more specific milestones may elicit a better response.

In the field of medical education, a Delphi consensus is a common way to determine components of a curriculum, develop assessment tools, and define competencies [27-28]. One of the main benefits of the online consensus group is that it has the potential to include a large number of participants from different and dispersed locations, improving feasibility. It is also inexpensive, anonymous, and limits the dominance of certain individuals that can disproportionately influence the group, avoiding direct confrontation of the experts [27]. However, this may limit the potential for discussion and debate [29].

Limitations of the study include low response rates, especially in the second round of the survey (6.55%), which may affect the generalizability of our results. This may in part be due to the online platform via email, which is often ignored. In the future, a similar survey may yield better response rates if a phone contact rather than email is used, especially if supported by a group with authority, such as the Royal College of Physicians and Surgeons of Canada. In addition, the survey would likely be better addressed to simulation or medical education experts, rather than program directors, which may yield a better response rate. Subgroup analysis based on demographics was not possible, given the small numbers, which may have introduced a geographical bias. We had 40 responses in part 1 and 15 responses in part 2. Group size in this setting does not depend on statistical power, but rather on group dynamics. Studies have shown that 10-18 participants in a Delphi study is recommended to reach conclusions and that the expertise of the panel may be more important than size [29-30]. Another limitation is missing data: some experts didn’t complete all sections. This was taken into account when calculating the means based on the number of responses for each item and not the total responses.

## Conclusions

In the last 15 years, there has been a reform in medical education with the integration of CBME for training. This model focuses on the learner and aims to ensure that all trainees graduate as competent physicians in all aspects of their specialty. With outcomes in mind, abilities, and competencies that each graduate needs are defined and developed into milestones and EPAs. Simulation will become an important and valuable tool in the training and assessment of residents in the CBD program, allowing for direct observation of skills. The results of this study indicate that most experts remain neutral about the role of simulation, except in the context of technical skills. However, this view will likely change as CBD becomes integrated into the curriculum, with more involvement of medical education experts.

With CBD just around the corner in Canada, all medical specialties, including OBGYN, will need to devise EPAs for each stage of residency and set benchmark levels for the trainees. These benchmarks will identify learners reaching milestones at varying speeds, allowing to tailor each learner’s needs individually as well as to identify those that would need additional help earlier on. An OBGYN national curriculum based on predefined EPAs and benchmark levels, as well as adequate assessment tools, including simulation, needs to be further explored for CBME to be successful. The results of this study can help inform future curricula.
